# (Not) Communicating the Environmental Friendliness of Food Packaging to Consumers—An Attribute- and Cue-Based Concept and Its Application

**DOI:** 10.3390/foods11091371

**Published:** 2022-05-09

**Authors:** Krisztina Rita Dörnyei, Anna-Sophia Bauer, Victoria Krauter, Carsten Herbes

**Affiliations:** 1 Institute of Marketing, Corvinus University of Budapest, 8 Fovam ter, 1093 Budapest, Hungary; krisztina.dornyei@uni-corvinus.hu; 2 Packaging and Resource Management, Department Applied Life Sciences, FH Campus Wien, University of Applied Sciences, Helmut-Qualtinger-Gasse 2/2/3, 1030 Vienna, Austria; anna-sophia.bauer@fh-campuswien.ac.at; 3 Institute for International Research on Sustainable Management and Renewable Energy, Nuertingen Geislingen University, Neckarsteige 6-10, 72622 Nuertingen, Germany; carsten.herbes@hfwu.de

**Keywords:** packaging, environmentally friendly, eco-friendly, sustainable, consumer, strategy, attribute, cue, marketing, wafer

## Abstract

While consumer understanding of and preferences for environmentally friendly packaging options have been well investigated, little is known about the environmentally friendly packaging attributes communicated to consumers by suppliers via packaging cues. We thus propose a literature-based attribute-cue matrix as a tool for analyzing packaging solutions. Using a 2021 snapshot of the wafer market in nine European countries, we demonstrate the tool’s utility by analyzing the cues found that signal environmentally friendly packaging attributes. While the literature suggests that environmentally friendly packaging is increasingly used by manufacturers, our analysis of 164 wafer packages shows that communication is very limited except for information related to recyclability and disposal. This is frequently communicated via labels (e.g., recycling codes, Green Dot) and structural cues that implicitly signal reduced material use (e.g., less headspace and few packaging levels). Our attribute–cue matrix enables researchers, companies, and policymakers to analyze and improve packaging solutions across countries and product categories. Our finding that environmentally friendly packaging attributes are not being communicated to consumers underscores a pressing need for better communication strategies. Both direct on-pack and implicit communication should help consumers choose more environmentally friendly packaging. Governments are encouraged to apply our tool to identify communication gaps and adopt labeling regulations where needed.

## 1. Introduction

From an environmental perspective, food packaging is both boon and bane. As a boon, it preserves food and supports its efficient transport; thus limiting the waste of food and resources [1,2,3,4]. However, the bane of packaging can seem overwhelming: nearly 200 kg of packaging waste is generated each year in the European Union per inhabitant [5]. A large part of that waste goes to incinerators or landfill [6], but much of the packaging ends up in the environment [7]. As the packaging market is expected to grow [8] and many of today’s packaging solutions are less environmentally friendly than they could be, both waste management and packaging systems call for redesign [9,10].

Packaging has become an environmental villain, a necessary evil, or even an unnecessary cost position that ought to be minimized [11,12]. The European Commission’s action plan for the circular economy aims at developing a sustainable, low carbon, resource efficient, and competitive European economy. Developing environmentally friendly packaging is one of the key items on its agenda [13], and thus a keen area of interest to scholars and practitioners [14,15].

Developing environmentally friendly packaging, however, is a difficult task. It is a balancing act between competing demands. Packaging must satisfy environmental requirements, food protection and logistics requirements, production and marketing requirements, and strategic and operational requirements. Such solutions cannot be developed by one company in isolation but only in the context of multidisciplinary product-packaging development teams [14]. These need to involve the entire supply chain: suppliers of raw material, products and packaging, brand owners, retailers, collectors, and recyclers [12].

It further complicates packaging development that consumers are not always eager to embrace environmentally friendly designs. Consumers perceive compromises between environmental friendliness and functional performance that they are unwilling to make [16,17]. Environmentally friendly packages are usually negatively associated with convenience, which leads to lower perceived functionality and a reduced willingness to purchase [16]. Consumers also harbor false beliefs about the benefits of alternative packaging materials (e.g., recycled, bio-based, or bio-degradable plastic) [17,18]. The willingness to purchase environmentally friendly packaging is further limited by time pressures and the cognitive overload caused by much information and a disinclination to process it [10]. In their defense, though, communication on sustainability is often misleading, which creates confusion and discomfort among consumers who are unable to differentiate between environmentally friendly packaging and packaging that just claims to be [19].

Still, for an environmentally friendly packaging solution to succeed in a consumer market, it has to meet with consumer acceptance. To better understand what that entails, this study adopts a consumer-based perspective on environmentally friendly packaging. That means we examine attributes and cues explicitly or implicitly perceived as sustainable by consumers.

Consumer perception does not necessarily agree with life cycle assessments (LCA), nor does it recognize the economic and social pillars of sustainability. However, it is critical for acceptance of a packaging solution. The fact is, consumers often harbor a simplified understanding of a packaging’s environmental impact and rely on behavioral routines and simple heuristics such as colors, material, or recycling options [10,20]. Additionally, sometimes they are outright wrong in their assumptions or evaluations of packaging [14,20,21,22]. That is why the consumer view of what makes a package environmentally friendly and how that friendliness can be recognized will not necessarily align with what is known by science of the environmental impact of a given solution [23].

This leads to a dilemma for packaging designers. If they base their design decisions solely on environmental assessments such as LCA, the design might fail in the market. However, if they base their designs on consumer perceptions, they might end up with environmentally inferior solutions. Hence, designers need to fulfill two objectives. First, packaging must fulfill engineering (or scientific) requirements. Designs must fit existing infrastructure (including machinery, available material, and food product needs), comply with changing regulatory environments, and have a comparatively low environmental impact as assessed by tools such as LCAs [22]. Second, packaging should communicate its benefits to consumers in a way that will be understood and recognized [10]. Thus, companies need to understand what consumers think makes a packaging solution environmentally friendly, i.e., the attributes and what they think and how they can recognize these attributes, i.e., the perceptual cues that signal environmental friendliness [23].

While research on environmentally friendly packaging has gained momentum with all stakeholders along the food and packaging supply chain [24], it has often focused on assessing environmental friendliness from a scientific view or from the demand-side perspective, i.e., consumer attitudes and perceptions or acceptance of environmentally friendly packaging solutions (e.g., ocean plastic). To the best of our knowledge, research has overlooked communication on the supply side, i.e., packaging choices available to consumers in the market. A well-structured mapping of packaging solutions in the market, one reflecting consumer perceptions of environmental friendliness, is needed to provide more effective messaging to consumers.

The present research aims at providing such a tool—an attribute–cue matrix combining two hitherto separate theoretical perspectives—to analyze packaging solutions across countries and product categories. To demonstrate the tool’s utility, we apply it to a snapshot of the wafer market as found in nine countries: Austria, Denmark, Finland, Greece, Hungary, Poland, Portugal, Slovakia, and Turkey. To the best of our knowledge, this field study is also the first comprehensive market analysis of environmentally friendly packaging communication in a specific product category across multiple countries.

How is our research useful for stakeholders in the packaging sector? First, while we apply the attribute–cue matrix to a sub-market of the food sector, it can be used to analyze any packaging solution in business-to-consumer (B2C) industries. That gives companies a practical tool to analyze their own packaging solutions and benchmark them against specific competitors or the industry. Any gaps derived from these analyses can be used as a point of departure for improving communication to the consumer at the point of sale.

Second, the attribute–cue matrix provides governments and regulators with a tool to survey how companies implement packaging solutions that claim to be environmentally friendly. Any inconsistencies found can then be addressed, if needed, by changes in regulations. The matrix also points out attributes and cues that a company does not yet use in its packaging solutions. These missed opportunities can serve as a starting point for new strategies, just as the matrix can help policymakers draft new regulations or project the impact of potential future regulations. The tool can also help environmental pressure groups and non-governmental organizations (NGOs) to document the state of packaging and communication approaches in specific industries and build their strategies from there.

Finally, for researchers in marketing and strategy, our combined conceptualization of attributes and cues provides a launch pad for cross-national and cross-industry studies of packaging strategies from the consumer perspective. This is especially important since a strong theoretical understanding of consumer perception of environmentally friendly packaging is still lacking [9,10,25] and existing knowledge is rather fragmented [15]. Therefore, we combine two theoretical perspectives: attributes that capture what consumers think makes a packaging solution environmentally friendly and cues, the core concept of cue utilization theory, that show how consumers think they can recognize these attributes. Moreover, we contribute to cue utilization theory by adding new cues that can be used in future research to analyze communication via packaging.

Our matrix, however, not only supports comparative research into strategies across countries, industries, or market segments on the supply side, but it also enables evaluating consumer attitudes and behavior against company strategies. This can potentially reveal gaps between the focus of a company’s messaging and what is important to consumers. Moreover, because we expand the concept of cues to include one sensory and three structural signals new to the literature, our attribute–cue matrix extends the strategic range of messaging to consumers.

The remainder of the paper unfolds as follows: in Section 2, we explain the theoretical foundations underlying our attribute–cue matrix. We then describe how we acquired empirical data from the multiple wafer markets and how we applied the matrix. In Section 3, we present our results. In Section 4, we discuss potential reasons for the packaging strategies found, after which we present implications for companies and policymakers. We conclude with avenues for further research.

## 2. Materials and Methods

### 2.1. Attribute–Cue Matrix

The attribute–cue matrix aims at identifying the messages cued by packaging that companies can use to communicate the environmentally friendly attributes of their packaging. The framework can be used both for analyzing consumer perceptions and behavior and for analyzing company packaging strategies. The concept of stimuli contents vs. formats that Ketelsen et al. have used for analyzing past studies (not products) ties in with our approach [15].

In its essence, the matrix combines packaging attributes that consumers perceive as environmentally friendly, e.g., biodegradability, with cues that explicitly or implicitly communicate the given attribute, e.g., a label indicating biodegradability. We derived both attributes and cues from previous consumer research on packaging perceived as environmentally friendly, using attributes proven to matter to consumers in their decision making. We also include attributes related to the efficient use of packaging, e.g., space-saving packaging, for the same reason.

From a consumer perspective, attributes are those characteristics that make a package environmentally friendly. These attributes can relate to various phases of packaging life: in raw material production, for example, consumers regard the use of recycled material or renewable material as environmentally friendly. In the post-use phase, consumers pay attention to biodegradability and recyclability. Table 1 summarizes these attributes, grouped by packaging life stages. The third column indicates previous studies that have shown the relevance of the respective attribute to consumer decision making. In the compilation of the attributes, we drew on Herbes et al. [21].

From a producer perspective, attributes describe packaging design choices, for example, the choice to use bio-based plastics for producing a pouch or to design the polymers for the pouch so they are bio-degradable. To communicate these attributes so they can enter into consumer purchasing decisions, designers need appropriate cues.

Cues are about communication. They are how companies communicate pro-environmental attributes of their packaging. This might be done by describing what part of the packaging is from a certain material, say ocean plastic. Cues describe how consumers recognize, or think they can recognize, pro-environmental attributes. Cues are necessary, because consumers often cannot experience directly the pro-environmental attributes of packaging. How, for instance, would a consumer know that the polymers for a pouch were bio-degradable? This is where cue utilization theory [45] comes in, when product characteristics cannot be objectively evaluated by observation. To reduce complexity, consumers make conclusions about products from the configuration of cues available [46]. Attributes that cannot be directly observed are called credence attributes [47]; for these, consumers have to trust the information provided by manufacturers on the package [48,49]. For example, the biodegradability of packaging is an attribute neither visible nor otherwise sense-perceptible. A consumer has to trust a manufacturer’s claim.

One attribute may be recognized through several cues. For example, consumers might think they can recognize renewable or recycled material by its color, but they may also look for a label or text on the packaging confirming the material’s origins.

Cues, however, can be treacherous if consumers have wrong ideas about packaging. Companies may deliberately mislead consumers by capitalizing on these wrong ideas, for example, using brown tones and coarse surfaces for packaging that is not from recycled or renewable material [15]. Some consumers, on the other hand, interpret pro-environmental cues as greenwashing, especially when claims diverge from expectations for environmentally friendly packaging design [25,28]. The multiple meanings of environmentally friendly packaging and the unclear packaging messages (e.g., labels) can create ambiguity, especially when environmental information is incorporated into a single metric or cue [50].

We chose to group environmental friendliness cues as experienced along the consumer journey: from first seeing the package at the point of sale, to then looking at the package closely, touching it and later, after the purchase, opening and using it (consumption). In the compilation of cues we drew on Herbes et al. [10].

We then added one new sensory cue and two new structural cues that consumers experience when using a product. They include, first, the sensory cue of how loosely or tightly a product is packaged, signaling how much packaging volume could have been saved. Next the product-to-packaging weight ratio, a structural cue, which though never measured directly by consumers does leave an impression. If the ratio is too low, consumers will read the cue as “overpackaged.” The second new structural cue we added is the number of packaging levels, which along with packaging waste pieces, is experienced directly by consumers when opening a product. The calculus of perception is as follows: the more levels, the more waste pieces, the less environmentally friendly.

We would like to point out that, in contrast to most other cues, these cues do not require a conscious marketing decision on the part of the manufacturer. Manufacturers may design lightweight packages (e.g., few packaging levels, few packaging pieces) for other reasons than consumer communication, such as savings in material or in logistic costs.

Table 2 presents the cues used in our analysis. These can all be found on or in the packaging itself, a constraint we imposed on our analysis since only these cues can be directly influenced by the manufacturer. Other cues consumers have been shown to use are the so-called social cues, information provided by retailers, friends, and family [10].

**Figure 1:**
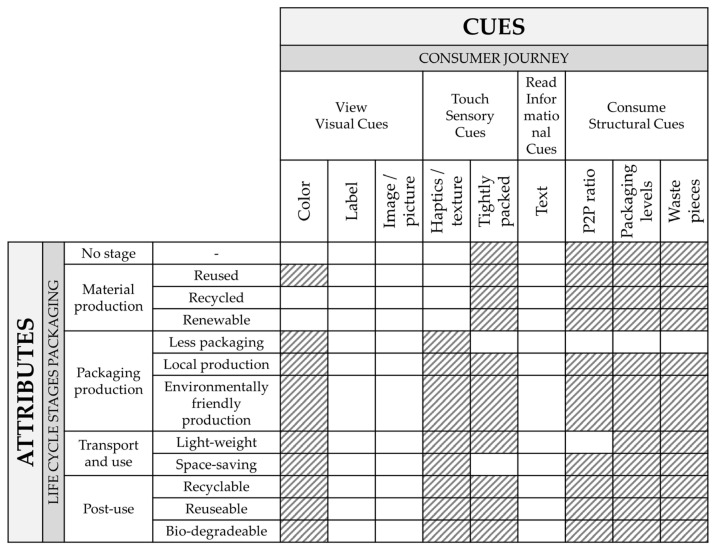
Attribute–cue matrix. Abbreviation: P2P ratio (product-to-packaging ratio).

Figure 1 presents the attribute–cue matrix, combining the attributes and cues described in Table 1 and Table 2. The matrix contains a total of 108 possible attribute–cue combinations, of which 49 are identified as practically applicable (colored white in Figure 1). For example, the fact that packaging is from renewable materials can be explicitly communicated through a label and text, and implicitly through images, surface texture, and color. Certain cues, such as labels, images, and text could be called all-purpose-cues, because they can be used to provide attribute-specific communication for all attributes. Other cues are more limited in their communication power; haptics for example, can be taken as a cue for renewable materials but not much else.

### 2.2. Sampling

To examine the environmentally friendly packaging options available to consumers in the market and to provide a snapshot of which messages about which attributes companies send to consumers through their packaging, a field study was conducted (with similarities to the field study of Deng and Srinivasan [58]). Wafer products were purchased from retail outlets to serve as data for the analysis.

Wafers are in the product group of cereals and confectionary; they were chosen for the study as a prime example of the impact that packaging can have on consumer decisions at the point of sale (POS). Among wafers, many different packaging options for similar products are available. The product category includes multiple sizes and packaging formats (types, material, shapes), as well as flexible packaging solutions such as fold wraps, flow packs, stand-up pouches and laminated paper bags, rigid plastic trays and boxes, metal-based boxes, and cardboard boxes.

Moreover, sustainable production and packaging of confectionery goods is a main area of interest for packaging redesign [59]. Sweets in general depend heavily on packaging [60] to take advantage of seasonal trade through colorful special editions. The main quality-related criteria for packaging confectionary products are protection against light, oxygen, and water vapor transmission [61]. To provide these high barriers, packaging designers often use material combinations that might yield non-recyclable packaging solutions [62]. However, the industry aspires to make progress in sustainable packaging. Indeed, an increasing number of news articles have appeared recently about the environmentally friendly aspirations of the confectionery industry [63].

The data collection portion of our field study ran from January to May 2021 in nine different countries—Austria, Denmark, Finland, Greece, Hungary, Poland, Portugal, Slovakia, and Turkey to cover as many products as possible. In each country, available packaging solutions in the wafer category were collected. Collections were made by a local researcher following these instructions: (1) define one shopping area (street, district, etc.); (2) within one week, visit all shops selling confectionary products in that area; (3) purchase all available wafer products (uncoated, chocolate, or nut-based filled wafers with at least two layers of wafers and one layer of filling); (4) repeat the shopping trip after 4–6 weeks to search for new products; and (5) send all (unopened) products accompanied by the shopping trip information to the research team members in Vienna for analysis. If researchers found the exact same packaging solutions in different “product series” of one brand with different sizes or fillings/flavors that matched the criteria, they were asked to purchase the cheaper option. This procedure resulted in a sample of 189 wafer products overall, of which 25 were excluded for being duplicates or not meeting the defined criteria for, i.e., flavor selection.

### 2.3. Analysis and Coding

Analysis of this data meant the careful examination of cues and the attributes companies communicate. The packaging examination was designed to best imitate the consumer journey and be as realistic as possible, so the analysis included not only the visual examination [64,65] but also the description (e.g., material, packaging type) and physical examination [66] of packages, including manually opening the packages. First, the content analysis [67] of packaging information was conducted; all environment-related textual and visual attributes were compiled in an Excel database. Second, the physical examination of packages was conducted, which included the opening, emptying, and exploring of disposal information of each package in a way that most closely resembles average product usage.

Coding used a combination of deductive and inductive approaches [67,68], since it started with environmental attributes and cues identified by previous research (deductive approach). Then during the analytical phase, new codes were added (inductive coding) to the category system—one sensory and two structural cues. One researcher coded the packages while two researchers assisted and revised coding to ensure objectivity and reduce rater bias. Codes were also re-examined by a fourth researcher, before the final coding scheme was developed (see Table A1 (Appendix A) for examples of coding rules).

After coding, the wafer data was processed through the attribute–cue matrix to obtain the frequency of use of each practically applicable attribute–cue combination. Based on these frequencies, we identified three main groups of cue usage. We then prepared the data for visual analysis using a heat map where cues used by the majority of products (≥50%) were marked red, cues used by a sizeable percentage (≥20%) were marked orange, and cues rarely used (<20%) were marked yellow. Other combinations, which were applicable but not used at all, remained white.

## 3. Results

**Figure A1:**
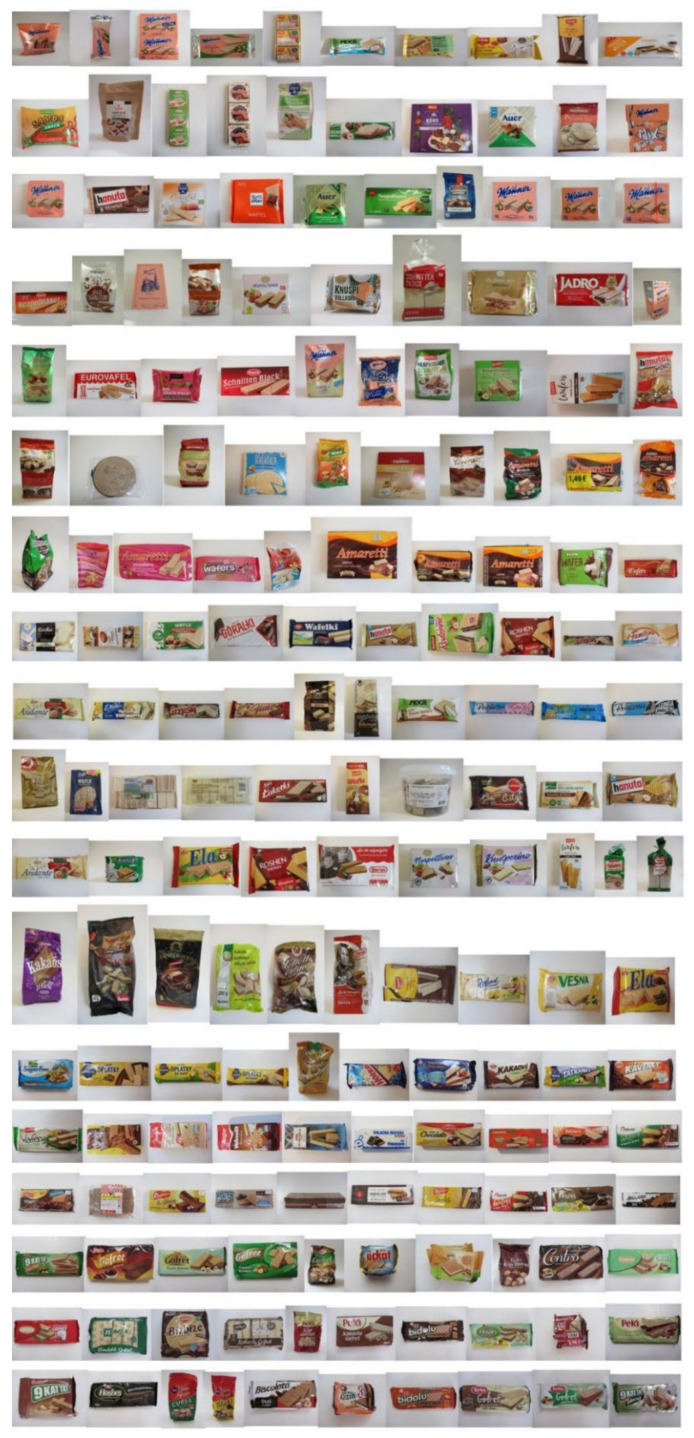
Pictures of all collected packages.

In total, 164 different wafer products were included in the analysis (see Figure A1 (Appendix B) for pictures of all collected packages, *n* = 189). The top three contributing countries for packages were Austria (33%), Turkey (20%), and Poland (13%). Other countries in the sample had shares of 10% or lower (*n* = 164, after discarding 25 as non-qualifying).

### 3.1. Descriptives

Flexible solutions were used by 88% of the products analyzed, whereas 11% of the products combined flexible (i.e., flow packs, fold wraps) and rigid elements, mostly plastic, rarely cardboard trays. Only one solution contained wafers as a bulk product in a solely rigid packaging solution, similar to a bucket with a lid and handle. Packaging made solely from plastic (excluding labels and clips) dominated the sample, making up 87% of the solutions. Information about the packaging being made from polypropylene (PP) and/or the recycling code/number five was frequently found. Only 13% of the packaging solutions included paper or cardboard elements, irrespective of labels including multilayer material (fold wraps, stand-up-pouches with paper layers) as well as boxes, trays, and inlays.

Referring to the surface haptics, 17% of the packaging surfaces were found to be coarse and/or matte as opposed to sleek and shiny. Investigating another sensory cue, the perception of excess air (headspace), found 79% of the solutions to be packed tightly, meaning the product could not move around in the package. Some solutions, such as trays in flow packs, were found to be intermediate (3%), i.e., between packed tightly and loosely. About one-fifth were packed loosely (18%).

The packages in the sample showed a variety of labels. Most of them related to the products, fewer to the packaging. One could find regional labels referring to local production, local certification schemes, as well as international certification standards commonly applied in the food production industry. Labels referring to certain ingredients, giving information about the cultivation or production of mostly cocoa, were frequently present. Labels relating to the packaging solution, e.g., the composition of the used materials and, less often, information about certified production standards in fiber-based solutions (paper, cardboard), for example, were found less often. Only one packaging solution in the 22 samples including paper carried a label related to agroforestry certification. Independent of the communicated material, the use of arrows arranged in a triangle or circle, with and without recycling code/number and the Green Dot, indicating collection or recycling context (76%), were found as well. Although symbols with recycling context/logos could help with correct post-use treatment by consumers, 39 of the collected packages did not contain the recycling code/number or a triangle/circle with arrows or the Green Dot on the outer packaging.

Surprisingly, text-based information referring to packaging was also quite rare (19 samples). Even though it is an all-purpose cue, text related to the packaging solution appeared on very few packages, stating, i.e., that the packaging solution is recyclable or that it is important to separate waste. On some packaging solutions one could find specific collection systems mentioned, i.e., for specific regions. Partly, the text-based information was available in combination or within a symbol, for example, stating in words which container to use for collection. These cases are reflected in the text-based share, not in the percentage of labels. More often, one could find, next to legally required labeling, information about the production, the ingredients and flavors, promotions or, for example, the brand values. As for the packaging solutions, the production or supplying company was communicated, but with logos rather than text. This was also the case for materials communicated as certified for food contact (FCM, fork, and glass). Moreover, none of the packages claimed to be bio-plastic/bio-based or of an environmentally friendly origin.

In terms of design, a total of 49 (30%) packages applied green as one of three main (most dominant) colors in the font of the brand name or the background color. If no brand name was found on the front of the pack, the product name was taken instead. Counting packages that were coded as being solely green, merely 7 (4%) of the wafers were found to have such a packaging design. Addressing images and pictures, one could find a multitude of different designs in backgrounds, brands, and product names on the wafer packages. Many of these images and pictures were, however, not found to be nature related (i.e., buildings, people, furniture, kitchen appliances, etc.) or, secondly, found to directly present the specific products (i.e., wafers), represent related processed ingredients (i.e., cocoa powder, chocolate, milk, cream, flour, etc.) and ingredient-related plants (i.e., hazelnuts, leaves of hazelnut trees, cocoa beans, cocoa plants, leaves of cocoa plants, vanilla blossom, ears of wheat, etc.). One could also find images and pictures of animals, but mostly cartoon style. All other additional images and pictures that were found to be nature-related (excluding the ones representing ingredients, animals, drop, and petal shapes), were rather limited and included trees, leaves and flowers, grass, mountains, landscapes, sun, moon, stars, clouds, etc. Counting only these, 18 (11%) packaging solutions carried one or more of such images or pictures.

The structural cue “product-to-packaging ratio” (written product weight versus emptied packaging) showed a broad range. The least efficient sample had a ratio of 1.75:1 whereas the most efficient solution had a rounded product-to-packaging ratio of 109:1. The most efficient solution was one package of 500 g wafers in a 4.6 g transparent flow pack. The sample’s average product-to-packaging-ratio rounded was 38:1, what was taken as a benchmark to identify the more efficient ones within the sample. In total, 79 (48%) packaging solutions had a higher ratio than this, meaning even higher efficiency, while the remaining 85 packages were less efficient.

Two other structural cues were investigated—the number of packaging levels (elements) that have to be opened to access the wafers, and the number of waste pieces of packaging that accumulate after consumption. Of the purchased products, 21% were multipacks with single packaged units (15% with 2–15; 4% with 6–10; and 2% with 11–25 pieces). However, only 15% of the purchased packages counted as having at least two levels to open. The difference between these two shares results from multipacks with single units that were held together by stickers, and therefore not considered as one level to open. The remaining 85% of packaging solutions required opening only one packaging element to access the wafers. Some solutions also included tear tapes/strips as well as text and/or graphic arrows to indicate where best to open the package.

The number of single packs and packaging levels goes hand-in-hand with the number of waste pieces generated by consuming the products. In 73% of the cases, only one piece of packaging waste accrued. Clearly, this number is smaller than that of levels to open, because partly open elements (such as trays) were counted as waste pieces, but not necessarily ones to open. Furthermore, opening multipacks was calculated as accessing one unit, which also accounts for the difference between waste pieces and levels to open. Only 2% of the packaging solutions produced more than 15 pieces of packaging waste; these cases were very small packages of less than 15 g of product.

### 3.2. Heatmap Based on the Attribute–Cue Matrix

**Figure 2:**
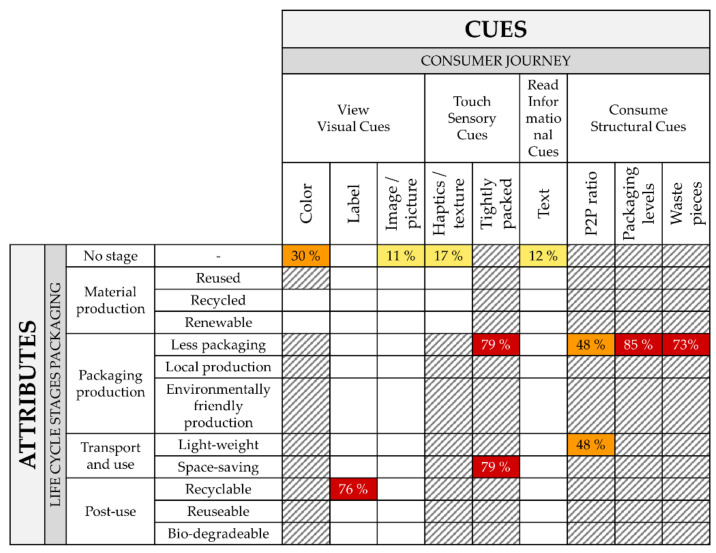
Heatmap of environmentally friendly cues that were utilized. Abbreviation: P2P ratio (product-to-packaging ratio).

Analyzing the wafer packaging data through the attribute–cue matrix yields the heatmap shown in Figure 2. Attribute–cue combinations that are not applicable appear as dashed cells, while practically applicable combinations not used appear in white. Of the 49 practically applicable attribute–cue combinations, only 12 (24%) were used by at least one product. Only four cues were hot (red ≥ 50%), with two cues lukewarm (orange ≥ 20%).

Traveling left to right in Figure 2, along the consumer journey, “color” was partially (≥20%) used, so it shows up orange. Labels were used more often, but primarily to indicate post-use: 76% of sampled packages carried labels relating to sorting or recyclability, reflecting recycling codes/numbers and the Green Dot. Images and pictures that communicate naturalness without any link to a specific stage in the packaging life were sufficiently present to move this cue from cold to cool, but still yellow in Figure 2.

Moving to the physical experience of the packaging, coarse and matte packaging textures, evoking a sense of naturalness, appeared as a cue with the same frequency category as images and pictures, leading to a similar yellow coding. The second sensory cue, “tightly packed”, was a hot signal for two different attributes (“less packaging” and “space-saving”). Text as an informational cue was used sparingly, leading also to its yellow coding.

Moving to the consumption phase, shown in the leftmost columns in Figure 2, more structural cues appear than in the other phases. This leads to more and hotter fields. A low number of packaging levels were used by around 85% of the packages. An optimized product-to-packaging ratio was found in more than 48% of the samples (the more efficient ones above average), producing the two orange fields and reflecting less packaging use in production and transportation.

The last structural cue along the consumer journey as well as the packaging’s life cycle stage, is given by accumulated waste pieces after consumption. This cue led to a hot field, as 73% of the packaging solutions only generated one piece of packaging waste.

Other cues were either not used or limited to a fraction of the products in the sample.

## 4. Discussion and Conclusions

This study developed a tool, the attribute–cue matrix, for analyzing the effectiveness of packaging solutions in communicating their environmental credentials to decision-making consumers. Only when consumers can recognize environmentally friendly packaging options will they be able to choose them. Without that demand-side perspective, even the best packaging solutions can go for naught.

We demonstrated the matrix through a field study of the wafer market in nine European countries—Austria, Denmark, Finland, Greece, Hungary, Poland, Portugal, Slovakia, and Turkey. While the matrix provides a powerful and versatile heuristic for academics, marketing managers, and policymakers, the results of the field study, based on 164 wafer packages, highlight more current topics relevant to communication and environmental specialists. The results show that even in the ever-popular wafer market, the supply side rarely communicates the potentially perceivable environmental attributes of its packaging solutions, compared to what would be possible.

These results are surprising, since environmentally friendly packaging is at the forefront of both academic and applied research. That it is not (yet) observable at the point-of-sale is thought-provoking, since here consumer perception of environmental friendliness and not the objective facts enter into a purchasing decision [69]. Our results are particularly sad given the gap between consumer perception of environmental friendliness and objective assessments of the life-cycle costs of a package [20,70]. This gap could narrow were effective guidance by unmistakable on-pack communication available to support pro-environmental product choices. That it is not in a popular mass market is puzzling.

In the next section we consider potential reasons for the puzzle. We then consider implications for companies and policymakers before outlining avenues for future research.

### 4.1. Potential Reasons for (Not) Communicating the Environmental Friendliness of Packaging Solutions

The first potential reason behind non-communicating lies in the properties of the product and the practical requirements of its packaging. Wafers are susceptible to water uptake (e.g., loss of crispness), are sensitive to oxidation (e.g., rancidity, unwanted color, and/or taste changes), can take up flavors and suffer structural damage [71,72,73] while having low water activity and therefore low susceptibility to the growth of pathogenic microorganisms. To extend the shelf life and the overall acceptability of wafers, producers opt for packaging solutions with high barriers against moisture, oxygen, light, and flavor loss. In many cases, it is difficult to meet all the packaging design requirements using a single material, so producers frequently opt for multilayer flexible food packaging solutions. These are built up of different materials that combine to meet functional requirements for i.e., resealability, barrier protection, strength, and lightweight, along with economic requirements for cost efficiency [74]. The latter also dictates minimal use of materials and often a reduced carbon footprint, both of which are environmental benefits. However, these materials show poor recyclability, a disadvantage heavily discussed as a trade-off among scientists and the public [62]. Therefore, even if a packaging solution is environmentally optimal for the product category, that fact is not easy to communicate.

The second potential reason for the dearth of effective on-pack communication can be found in the role the product plays for consumers and the context in which wafers are consumed. The consumption of confectionary products is often driven by hedonic motives [75], and it still relies on classic impulse triggers. Being reminded of one’s responsibility for the waste generated by the package, being beleaguered by details on the environmental impact of the packaging could have a sobering effect on a consumer, perhaps prompting second thoughts that would undermine the sale.

The contradiction between hedonic motives and moral choices is well-known in the literature [76] and most probably not from a perspective appealing to manufacturers of confectionaries. Manufacturers may not want to suppress hedonic impulses with environmental friendly packaging claims or to place moral principles over pleasure [76], because sustainability-linked attributes can affect hedonic properties negatively [77]. However, it is also possible for consumers to derive pleasure from doing something positive for the planet (see the concepts of ‘alternative hedonism’ [78,79] or ‘warm glow’ [71]), but this concept is probably difficult (though not impossible) to apply to environmentally friendly packaging of confectionaries. Still, despite extensive academic discourse on the dichotomy between hedonism and morality in consumption practices, we do not know what role these concerns played in the decisions made by the companies. How companies go about meeting both business and ethical obligations becomes a question for further research.

A third way to look at (the lack of) manufacturers’ on-pack communication strategies is through the model of ecological responsiveness [80], which names three motives for companies to behave pro-environmentally: to improve competitiveness, to create legitimation, and to fulfill a sense of responsibility to the earth. All three goals can be advanced by environmentally friendly packaging, a straightforward example being the competitive edge gained by saving resources and waste and streamlining logistics [81,82]. However, the development of such packaging entails high production costs, slow time-to-market, technical difficulties, and complex cross-team alignments [14]. Many times, companies lack the business expertise or long-term planning horizon needed to pursue eco-friendly packaging [21]. This is especially true in a product category not under criticism. As it is, businesses are often compelled by law to adopt environmentally friendly packaging initiatives (the legitimation motive) [14,25], but maybe not yet pressing over all product categories.

The fourth potential reason behind scarce on-pack communication is the novelty of the topic. Communicating the environmental friendliness of packaging is just beginning, especially when compared to product related on-pack information (e.g., organic labels or health claims), which have been hotly debated for decades and have evolved from the nonregulatory action policy of a few selected companies to a heavily regulated area [83].

### 4.2. Implications for Companies and Policymakers

How can scholars, managers, and policymakers use our research and what can be gained from it? This section advances implications aimed at addressing the key issues in relation to environmental packaging management, to stimulate greater attention to this important topic and to expand the scope of discussion.

The tool we have demonstrated provides guidance to companies considering environmentally friendly packaging communication. The attribute–cue matrix summarizes and visualizes the attribute–cue combinations that manufacturers may use. The matrix can help evaluate the status quo, compare competitive offerings, analyze potential communication directions, and improve existing packaging solutions.

Furthermore, the matrix can be used to improve packaging design: both communication changes and structural design changes can emerge from applying it. While considering packaging redesign, companies need to consider questions such as: How are consumers making sense of the current on-pack communication? Do they want to make a well-founded choice decision prioritizing certain eco-friendly attributes over others? How do consumers make sure that they recognize these attributes from the cues on the packaging across products from different manufacturers? And how can consumer perceptions be aligned with objective environmental impact?

Besides that, our results also indicate that both direct on-pack and implicit communication should be used more often to inform consumers and allow them to choose environmentally friendlier packaging solutions. Companies can use the matrix to identify better ways to provide this information [70] and explicitly signal the package attributes that qualify as environmentally friendly—especially compared to competitors. Using multiple signals of environmental friendliness is supported by cue congruence theory.

This study also provides guidance to policymakers. Our results show that with absent regulation, packaging communications can run the gamut, presenting the consumer with a cacophony of different messages from different producers, each highlighting different attributes with different cues. This more often creates misunderstanding and confusion for the consumer than providing real help in making pro-environmental purchase decisions. As in other markets for eco-friendly products, such as the markets for green electricity or for eco-friendly food, there is a potential positive role for a standardized, easy-to-understand information system, possibly administered by the state. However, the agonizing discourse and stubborn resistance from manufacturers over the nutriscore front-of-pack labeling [84] of food in Germany, France, and other countries [85,86,87,88] shows how difficult it is for policymakers to establish such a system. However, with sustainability-related credence attributes gaining more and more importance and consumers being less and less able to judge products with their five senses, accurate and informative labeling becomes a key task for third party actors such as industry associations or the state.

Both policymakers and manufacturers should consider the lack of communication about the end of life of packages. Not only is there almost no on-pack information to help consumers dispose of the package, but even if there were, the collection system in Europe varies from country-to-country and in some countries by region. Perceivable cues on products sold in multiple European countries would have to include regional labeling, which simply is not feasible. Therefore, it appears that action is still needed to reach the recyclability goals of the European Plastic Strategy by 2030 [89] and to ensure that improvements align with the overall goal of sustainability.

### 4.3. Avenues for Further Research and Limitations

This study is not without limitations and our work hints at multiple avenues for future research. First, we demonstrate a versatile and powerful tool, but do so considering only packaging from one product category in nine countries. Undoubtedly, a larger and more heterogeneous sample would provide a richer understanding of current on-pack communications and might even expand the tool, as applied packaging solutions could differ from the ones found in the category of confectionary products. The validation or further development of the matrix with different sample sets would be beneficial to check for differences across product groups. Therefore, we recommend the attribute–cue matrix be used in the analysis of packaging strategies across product categories and markets, where large differences can be expected due to different consumption factors or packaging solutions.

Second, it would be helpful to understand why companies design packaging solutions the way they do and why they do (not) communicate the way we might think they should. Do restrictions stemming from technical properties of packaging material and machinery as well as requirements of packaged products largely govern packaging solutions? How do companies position the environmental friendliness of packaging solutions in their marketing strategies? Which stakeholders inside and outside the company are involved in packaging design decisions? How do companies see their potential customers and how do they think customers factor environmental issues of packaging into their buying decisions? These questions call for a qualitative study of decision-making processes involved in packaging design in companies.

Third, let us turn from the supply side to the demand side. Largely absent from the literature are comparative studies of consumer preferences for environmentally friendly packaging across product categories. Do consumers have different preferences regarding pro-environmental attributes of packaging and are they receptive in different ways to cues communicating these attributes depending on the product category and the consumption context? The discourse on the relationship between hedonism and sustainable consumption suggests that environmental impact may be less of a concern for consumers when the product and its consumption are embedded in hedonism.

Another question is which cues are especially credible and effective in communicating pro-environmental attributes. We hypothesize that some attributes would best be communicated by text, others best by nontextual cues. Lastly, it would be helpful to understand how consumers examine a package to determine its environmental friendliness. Observations and eye tracking could be suitable methods to explore this.

Answering these questions would help companies better understand how they can build pro-environmental considerations into their packaging strategies and how they might better help consumers make sound pro-environmental choices. Pursuing these questions would also help policymakers understand where consumer preferences, even if understood well by companies, cannot drive improvements in the overall sustainability of packaging solutions and where, therefore, a positive role for regulation may exist.

## Figures and Tables

**Table 1:** Pro-environmental attributes of packaging solutions.

Stage in Packaging Life	Pro-Environmental Attribute	Source
*Material production*	Reused packaging	[26,27]
	Recycled materials	[18,19,28,29,30]
	Renewable materials (bio-based)	[18,20,22,23,25,31,32,33]
*Packaging production*	Less packaging	[34,35,36,37,38]
	Local/regional production	*
	Environmentally friendly production	[25]
*Transport and use*	Lightweight	*
	Space-saving	[39]
*Post-use*	Reusable	[23,25,26,40,41]
	Recyclable	[19,23,25,29,30,35,42,43]
	Bio-degradable	[23,25,29,30,40,44]
*General (no specific stage)*	Environmentally friendly in general	[40]

* newly proposed attributes.

**Table 2:** Cues on pro-environmental attributes of packaging solutions.

Consumer Journey	Cue Type	Cue	Source
Point of Sale	Visual (from distance)	Color	[10,39,40]
		Label/logo	[10,39,51,52]
		Image/picture	[22,53,54]
	Sensory (touching/picking up)	Haptics/texture/material	[10,12,22,25,55,56]
		Loose/tight packaging	*
	Informational (reading)	Text	[10,27,28,39,53,54,57]
Consumption	Structural (use-phase)	Product-to-packaging ratio	*
		Number of packaging levels	*
		Number of packaging waste pieces	[10]

* newly proposed cues.

## Appendix A

**Table A1:** Examples of coding rules for the analysis.

Cue	Coding Rule: Coded as Signaling Environmental Friendliness if …	Example
Color	Packaging was any shade of green	
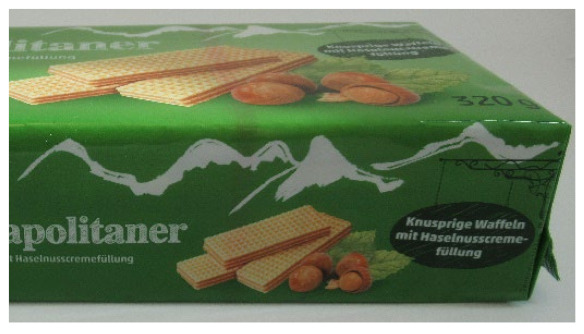
Label	Recycling code and/or symbol and/or green dot was present	
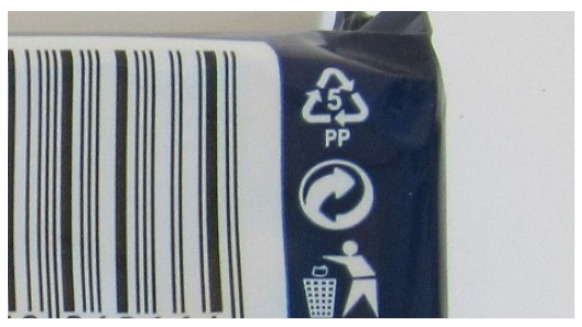
Image/picture	Nature-related images were present. We excluded any nature-related pictures or graphics that had a direct link to the product and its ingredients, e.g., a cocoa tree	
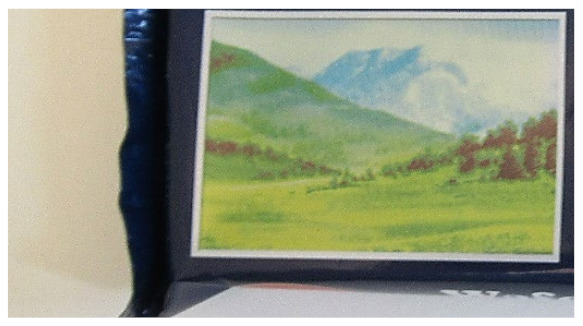
Haptics/texture	Material was coarse or matte	
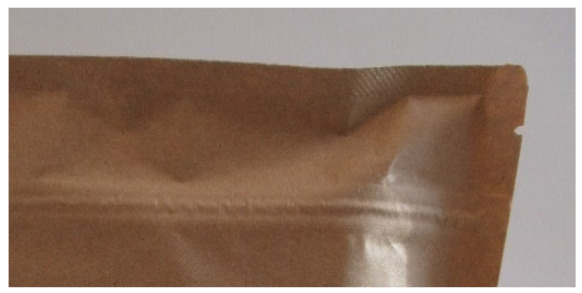
Tightly packed	Product was tightly packed (minimum headspace)	
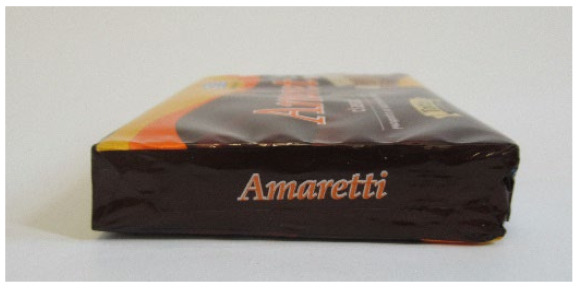
Text	Information on environmental attributes of the packaging was present, e.g., general ecological benefits, appeals for waste treatment or reduction in greenhouse gas emissions	
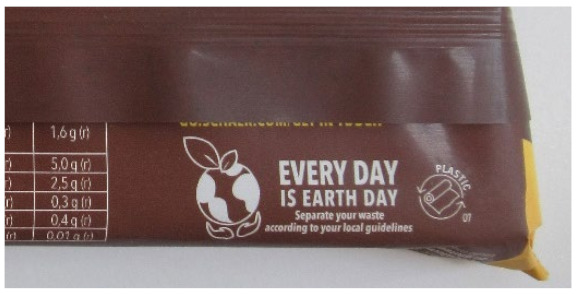
Weight of the product relative to packaging weight	High product-to-packaging ratio	
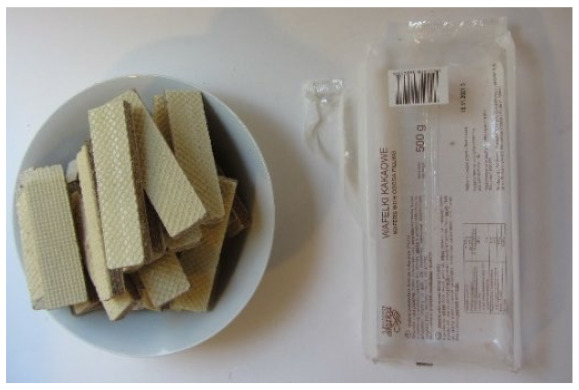
Number of packaging levels	No more than one level to open	
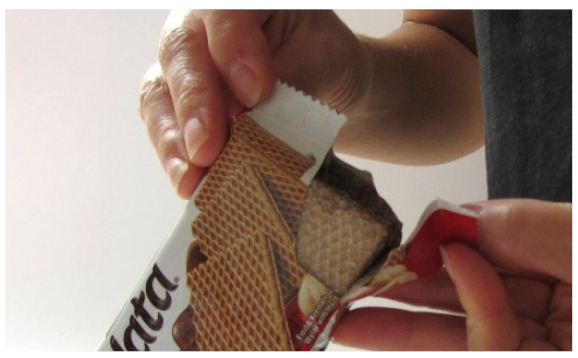
Number of packaging waste pieces	Only one waste piece	
